# Ventilatory support and inflammatory peptides in hospitalised patients with COVID-19: A prospective cohort trial

**DOI:** 10.1371/journal.pone.0293532

**Published:** 2023-11-02

**Authors:** Maximilian Robert Gysan, Christopher Milacek, Christina Bal, Andreas Zech, Jonas Brugger, Ruxandra-Iulia Milos, Lukasz Antoniewicz, Marco Idzko, Daniela Gompelmann

**Affiliations:** 1 Division of Pulmonology, Department of Internal Medicine II, Medical University of Vienna, Vienna, Austria; 2 Institute for Medical Statistics, Medical University of Vienna, Vienna, Austria; 3 Department of Biomedical Imaging and Image-guided Therapy, Medical University of Vienna, Vienna, Austria; Children’s National Hospital, George Washington University, UNITED STATES

## Abstract

**Purpose:**

Several studies have shown that SARS-CoV-2 can induce a massive release of cytokines which contributes to disease severity and mortality. Therefore, cytokine levels in the serum may help to predict disease severity and survival in COVID-19 patients.

**Methods:**

In this prospective trial, 88 patients who were hospitalised for COVID-19 were enrolled. Blood samples for serum peptide measurements were taken at the time closest to hospitalisation, at day 5, 9 and 13 (±1). The concentrations of cytokines (IL-1α, IL-1β, IL-1RA, IL-6, L-7, L-10, IFN-γ and TNF-α), chemokines (CCL-3, CCL-4 and CCL-7) and growth factors (G-CSF, GM-CSF and VEGF) were assessed and correlated with the type of ventilation, occurrence of consolidations on imaging and the level of care.

**Results:**

COVID-19 patients (median age 68 years, IQR 55–77) stayed in hospital between 5–171 days. Compared to patients in the general care unit, patients in the intermediate care unit (IMCU) and intensive care unit (ICU) presented significantly elevated serum IL-6 (p = 0.004) and lower IFN-γ levels (p = 0.005), respectively. The peak inspiratory pressure in ventilated patients correlated positively with IL-1RA, G-CSF and inversely with IFN-γ serum levels (all p<0.05). VEGF serum levels inversely correlated with the fraction of inspired oxygen in patients receiving high-flow nasal canula oxygen therapy (p = 0.047). No significant correlation between serum concentrations of the measured peptides and the type of ventilation, occurrence of radiological consolidations or in-hospital mortality has been observed.

**Conclusion:**

IL1-RA, IL-6, IFN-γ, G-CSF, CCL-7 and VEGF serum levels could prove helpful as biomarkers to assess disease severity and the need for intensive care in COVID-19 patients.

## Introduction

In December 2019, SARS-CoV-2 (severe acute respiratory coronavirus 2) was identified for the first time in Wuhan, Hubei Province, leading to COVID-19 (coronavirus disease 2019). It is the cause of the COVID-19 pandemic, which was declared a public health emergency of international concern by the WHO on 30th January 2020 and a pandemic on 11th March 2020.

SARS-CoV-2 infection is associated with mild flu-like symptoms in 80% of infected subjects. During the initial phase of the pandemic, it was estimated that up to 20% of cases were severe and required hospitalisation. Overall, 5–14% of patients required ICU treatment, out of which approximately one quarter died [1–3]. Severe COVID-19 may be caused by a cytokine storm due to an excessive immune reaction in response to the angiotensin-converting enzyme 2 (ACE2)-mediated entry of SARS-CoV-2 into cells [4]. Cytokine release can be fuelled by IFN and NF-κB production which leads to the homing of a variety of immune cells to the site of infection contributing significantly to the pathogenicity of SARS-CoV-2 by destabilizing endothelial cell to cell interactions, damaging vascular barriers and causing capillary and diffuse alveolar damage [5–8].

There are already numerous studies showing that inflammatory cytokines such as IL-1, IL1RA, IL-2, IL-4, IL-6, IL-7, IL-8, IL-9, IL-10, IL-12, IL-13, IL-17, basic FGF, GCSF, GMCSF, IFN-γ, IP-10, MCP-1, MIP-1A, CCL-4, PDGF, TNF-α and VEGF can be detected in COVID-19 plasma samples [9–12]. This cytokine storm can lead to respiratory failure, shock and multi-organ failure [9, 13–15]. Huang *et al*. demonstrated that COVID-19 patients who required intensive care due to respiratory failure had higher plasma concentrations of IL-2, IL-7, IL-10, GCSF, IP-10, MCP-1, CCL-3 and TNF-α than non-intensive care patients [14]. The clinical significance of the cytokine storm in hospitalized severe COVID-19 cases was highlighted by Chen *et al*., who showed that patients with severe disease defined as respiratory rate > 30 per minute, percutaneous oxygen saturation of 93% or lower or need for high flow nasal cannula (HFNC) or non-invasive ventilation (NIV) had higher levels of IL2-R, IL-6, IL-10 and TNF-α [9]. Similarly, elevated levels of IL-6, IL-10, IL-2 and IFN-γ were found in patients with severe COVID-19 due to respiratory failure [15]. Thus, cytokines appear to be predictors of disease severity. Most of the previously published data were retrospective and cross-sectional studies, analysing cytokines at single timepoints. Only a few observational studies were conducted so far. The objective of this study was to assess serum concentrations of cytokines, chemokines and growth factors during the course of disease and to evaluate their ability to predict disease progression in COVID-19 patients.

## Methods

### Study design

In this prospective observational cohort study, we included patients with COVID-19 who were hospitalised at the Division of Pulmonology of the Vienna General Hospital between 06.01.2021 and 31.05.2021. Inclusion criteria were patient age over 18 years, SARS-CoV-2 infection confirmed by a positive polymerase chain reaction (PCR) and signed informed consent. The exclusion criterium was withdrawal of consent to participate in the study.

Patients demographics, onset and nature of symptoms, comorbidities, relevant laboratory values and medical management data were obtained. Disease severity was assessed at admission using the WHO Clinical Progression Scale and the 4C-Score [16, 17]. Medical management data included ventilation, findings on chest X-ray or if available multi-detector computed tomography scan (MDCT), medication and level of care. Blood samples for the measurement of serum peptide concentrations were collected at the time closest to hospitalisation and repeated at day 5, 9 and 13±1. Complete blood counts were drawn along with the sample for the first serum peptide measurement ±2 days.

In order to correlate serum concentrations of cytokines, chemokines and growth factors with disease progression in patients with COVID-19, we assessed the ventilatory support, radiological findings and level of care. Ventilation was defined as nasal oxygen therapy, high flow nasal cannula (HFNC), non-invasive ventilation (NIV) or mechanical ventilation (MV). Non-invasive ventilation included continuous positive airway pressure and bilevel positive airway pressure. Every patient received a chest X-ray or MDCT of the chest upon hospital admission. These assessments were repeated when clinically indicated. The course of radio-morphologic findings was defined as regressive, stable, or progressive by a radiologist, who was blinded for the other outcome parameters. Level of care and disease progression were defined using the WHO Clinical Progression Scale and the 4C Score [16, 17]. Patients with moderate disease requiring no oxygen or nasal oxygen therapy according to a WHO clinical progression scale of 4–5 received general care. Patients with severe disease requiring HFNC or NIV according to a WHO clinical progression scale of 6 received intermediate care on an intermediate care unit (IMCU). Patients with severe disease requiring intubation and mechanical ventilation according to a WHO clinical progression scale of 7–9 received intensive care on an intensive care unit (ICU). Further, we assessed the correlation between serum peptide levels and in-hospital mortality as well as length of hospital stay.

The ethics committee of the Medical University of Vienna approved the protocol of this study (2355/2020) and all patients gave written informed consent prior to study enrolment.

### Assessment of blood samples

Cytokines, chemokines and growth factors were assessed using enzyme-linked immunosorbent assays (ELISA). Blood samples were collected in serum collection tubes, inverted 10 times and incubated at room temperature for 30–60 mins. Before sera were obtained, the tubes were centrifuged with 1200 x g for 15 min. Samples were frozen at -80°C until measurement of serum peptide levels using ELISA DuoSets (R&D Systems, MN, USA) according to manufacturer’s manual. The applied immunoassays with the corresponding detections limits are listed in S1 Table. In addition, routine laboratory parameters including blood counts, c-reactive protein, creatinine and blood urea nitrogen (BUN) were assessed using automated analysers.

### Statistics

Continuous variables are reported as mean with standard deviation or median with range or interquartile range. Categorical variables are reported as frequency. We fitted separate linear mixed models with the respective logarithmic serum peptide concentration as the dependent variable and ventilation modality, radiological findings and level of care as the explanatory variable. We then conducted likelihood ratio tests on the explanatory variables and performed post-hoc comparisons in case the likelihood ratio test was statistically significant. For post-hoc comparisons regarding the level of care, we chose the general unit as the reference group. The serum concentrations of peptides were correlated with the peak ventilation pressure in patients receiving NIV or MV and with the oxygen demand (defined as the fraction of inspired oxygen, fiO_2_) in patients receiving NIV, MV or HFNC. For this purpose, we fitted linear mixed models with the logarithmic cytokine concentration as the dependent variable and the peak ventilation pressure or fraction of inspired oxygen as the explanatory variable. Separate models were calculated for each serum peptide. All of the linear mixed models were adjusted for time of blood draw and include a random effect for the patient. We considered only logarithmic serum peptide concentrations to better fulfil the normality assumption of the linear model. For the logarithm to be defined, all cytokine concentrations were increased by 1. To evaluate the association of cytokine concentrations and the in-hospital mortality, we applied univariable cox regression models. The models were adjusted for age and sex of the patient. The time of survival was defined as the time between hospital admission and either discharge or death. The serum peptide concentrations, which were measured on multiple time points, were treated as time-dependent covariates in the cox regression analysis. The association of serum peptides and length of hospital stay was evaluated applying fitted linear mixed models. P-values below 0.05 were considered statistically significant. However, no correction for multiple testing was performed, therefore p-values are of descriptive nature. Statistical analysis was performed using R version 3.6.1 or higher.

### Ethics approval

This study was performed in line with the principles of the Declaration of Helsinki. Approval was granted by the Ethics Committee of the Medical University of Vienna (Ethics Vote No. 2355/2020).

## Results

### Study population

In total, 88 patients were enrolled in this prospective trial within 4.2±3.5 days after hospital admission. Patient characteristics are presented in Table 1. The median age was 68 years (19–94) and 69.1% of the participants were male (n = 61). The most frequent comorbidities were cardiovascular disease (71.6%, n = 63) followed by diabetes (25%, n = 22) and kidney disease (25%, n = 22). Table 1 provides an overview of the pre-existing comorbidities. 10 patients received immunosuppressive therapy after transplantation of the kidney (n = 6), lung (n = 2), liver (n = 1) or heart (n = 1). 1 patient was immunodeficient due to HIV.

**Table 1:** Patient demographics.

Variable	n = 88
Age, median (range)	68 (19–94)
Sex	
Male	61 (69.1%)
Female	27 (30.7%)
Comorbidities	
Cardiovascular disease	63 (71.6%)
Arterial Hypertension	41 (46.6%)
Arrhythmia	28 (20.4%)
Coronary artery disease	15 (17.1%)
Peripheral artery disease	12 (13.6%)
DVT and Pulmonary embolism	9 (10.2%)
Congestive heart failure	8 (9.1%)
Valvular disease	6 (6.8%)
Pulmonary hypertension	3 (3.4%)
Congenital heart disease	1 (1.1%)
Neurologic disease	16 (18.2%)
Stroke or transient ischemic attack	10 (11.4%)
Parkinson’s disease	3 (3.4%)
Depression	2 (2.3%)
Polyneuropathy	2 (2.3%)
Dementia	1 (1.1%)
Congenital disorder	1 (1.1%)
Epilepsy	1 (1.1%)
Respiratory disease	15 (17%)
COPD	9 (10.2%)
Asthma	2 (2.3%)
Interstitial lung disease	2 (2.3%)
Autoimmune disease	6 (6.8%)
Polymyalgia rheumatica	2 (2.3%)
Granulomatosis with Polyangiitis	1 (1.1%)
Crohn’s disease	1 (1.1%)
Rheumatoid Arthritis	1 (1.1%)
Systemic Lupus erythematosus	1 (1.1%)
Hepatic disease	5 (5.7%)
Fatty liver disease	2 (2.3%)
Hepatitis B	2 (2.3%)
Cirrhosis	1 (1.1%)
Diabetes	22 (25%)
Kidney disease	22 (25%)
Chronic renal insufficiency of unknown cause	17 (19.3%)
Nephrolithiasis	2 (2.3)
IgA Nephritis	1 (1.1%)
Hypertensive kidney disease	1 (1.1%)
Pyelonephritis	1 (1.1%)
Malignancy	19 (21.6%)
Colorectal cancer	4 (4.6%)
Lymphoma or Leukaemia	3 (3.4%)
Prostate cancer	3 (3.4%)
Breast cancer	2 (2.3%)
Anal cancer	1 (1.1%)
Pancreatic cancer	1 (1.1%)
Lung cancer	1 (1.1%)
Bladder cancer	1 (1.1%)
Melanoma	1 (1.1%)
Thyroid cancer	1 (1.1%)
Prolactinoma	1 (1.1%)
Cancer of unknown primary	1 (1.1%)
WHO Clinical Progression Scale, median (range)	5 (4–6)
4C Score, median (IQR)	9 (6–12)
Days from symptom onset to hospitalisation, mean (range)^†^	5.1 (0–28)
Days from hospitalisation to first blood draw, mean (range)	5.0 (0–41)
Secondary infections requiring antibiotic treatment	63 (71.6%)
Hospitalisation in days, median (range)	15 (5–171)
In-hospital mortality	18 (20.5%)

† 4 patients were hospitalized prior to diagnosis of COVID-19

Common symptoms were dyspnoea (47%, n = 41), cough (57%, n = 50), fever (33%, n = 29), diarrhoea (25%, n = 22), chest pain (5%, n = 4) and dysgeusia/ dyssomnia (9%, n = 8). 83% of patients received dexamethasone and 72% required antibiotics for the treatment of secondary infections. 38% of patients (n = 33) were hospitalised in the general unit, whereas 57% (n = 50) required IMCU treatment and 6% (n = 5) ICU treatment. The majority of patients showed consolidations on chest X-ray or MDCT (85.2%, n = 75). 44% of patients (n = 39) received NIV or MV, 11% (n = 10) received HFNC and 32% (n = 28) received supplementary oxygen. The remaining 11 patients received no oxygen during hospitalisation (Table 2). In-hospital mortality was 20.5% (n = 18).

**Table 2:** Course of ventilation, radiological findings, level of care and serum peptide values.

Variable	Day 1	Day 5	Day 9	Day 13
	(n = 88)	(n = 80)	(n = 54)	(n = 37)
**Ventilation**				
**no supplementary oxygen**	25 (28.4%)	26 (32.5%)	19 (31.2%)	13 (35.1%)
**supplementary oxygen**	35 (39.8%)	27 (33.8%)	15 (27.8%)	10 (27%)
**HFNC**	7 (8%)	7 (8.8%)	2 (3.7%)	3 (8.1%)
**NIV**	21 (23.9)	19 (23.8%)	16 (29.6%)	11 (29.7%)
**MV**	0	1 (1.3%)	2 (3.7%)	0
**Radiological findings**				
**no consolidations**	25 (28.4%)	4 (5%)	4 (7.4%)	0
**consolidations**	61 (71.6%)	32 (40%)	24 (44.4%)	29 (54.1%)
**Level of care**				
**general unit**	56 (63.6%)	49 (61.3%)	34 (63.0%)	22 (59.5%)
**IMCU**	32 (36.4%)	29 (36.3%)	16 (29.6%)	12 (32.4%)
**ICU**	0	2 (2.5%)	4 (7.4%)	3 (8.1%)

HFNC: high flow nasal canula, NIV: non-invasive ventilation, MV: mechanical ventilation, IMCU: intermediate care unit, ICU: intensive care unit

### Serum concentrations of different peptides

Among most of the patients, serum levels of IL-1α, IL-1β, IL1-RA, IL10, G-CSF, GM-CSF, IFN-γ, CCL-7, and TNF-α were low or below the detection limit at different time points of measurement. Concentrations of IL1-RA and CCL-3 varied substantially. The progression of serum peptides is shown in S1 Fig. Levels of IL-1 ß, IL-1 RA, G-CSF, CCL-3, TNF-α and VEGF decreased during the observation period while levels of IFN-γ increased. Levels of IL-1 α, IL-6, IL7, IL10, GM-CSF, CCL-4, CCL-7 remained mostly unchanged.

### Correlation of serum peptides and ventilation, radiological findings, level of care, in-hospital mortality and length of stay

**Fig 1:**
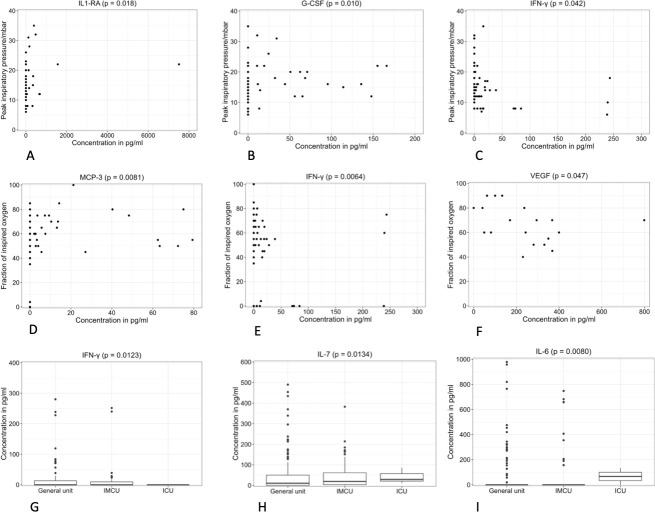
Correlation of serum peptides, ventilation and level of care. a-e: Correlation between serum peptides and ventilatory support in patients receiving NIV or MV: Peak inspiratory pressure (a-c), Fraction of inspired oxygen (d-e), f: Correlation between serum peptides and patients receiving HFNC: Fraction of inspired oxygen, g-i: Correlation between serum peptides and level of care depicted as boxplots. IL-1 RA: interleukin-1 receptor antagonist, G-CSF: granulocyte colony-stimulating factor, IFN-γ: interferon γ, CCL: CC-chemokine ligand.

IL1-RA, IL-6 and IFN-γ concentrations correlated with the level of care (Fig 1). IL-6 levels were about 2.2 times higher in patients in the IMCU compared to patients in the general unit (p = 0.004), IFN-γ levels in ICU patients were only a quarter of the IFN-γ-levels of patients in the general unit (p = 0.005) (Table 3). Furthermore, there was a trend towards higher IL1-RA-levels in patients in the ICU compared to patients in the general unit (estimate [95% CI]: 4.28 [0.89; 20.55], p = 0.07).

**Table 3:** Association between serum peptide levels and ventilatory support.

	Ventilation pressure in patients with NIV or MV.		Oxygen demand in patients with NIV or MV		Oxygen demand in patients with HFNC	
**Variable**	**Estimate [95% CI]**	**p-value**	**Estimate [95% CI]**	**p-value**	**Estimate [95% CI]**	**p-value**
**IL-1 α**	1.05 [0.98–1.12]	0.187	1.00 [0.98–1.02]	0.959	1.0 [0.99–1.01]	0.828
**IL-1 β**	1.03 [0.96–1.10]	0.423	1.00 [0.98–1.02]	0.951	1.0 [1.0–1.0]	0.617
**IL-1 RA**	**1.15 [1.03–1.29]**	**0.018**	1.00 [0.98–1.03]	0.783	1.00 [1.00–1.00]	0.622
**IL-6**	1.06 [0.96–1.16]	0.244	1.01 [0.99–1.04]	0.206	1.01 [0.96–1.06]	0.733
**IL-7**	0.99 [0.95–1.04]	0.783	1.00 [0.99–1.01]	0.461	0.97 [0.93–1.01]	0.220
**IL-10**	1.02 [0.95–1.10]	0.591	1.01 [0.99–1.03]	0.40	0.96 [0.90–1.02]	0.170
**G-CSF**	**1.10 [1.03–1.19]**	**0.010**	1.01 [0.99–1.03]	0.371	0.96 [0.89–1.03]	0.278
**GM-CSF**	1.01 [0.94–1.09]	0.790	1.00 [0.99–1.02]	0.61	0.95 [0.90–1.01]	0.147
**IFN-γ**	**0.92 [0.86–0.99]**	**0.042**	**0.98 [0.96–0.99]**	**0.006**	0.99 [0.95–1.05]	0.733
**CCL-7**	1.00 [0.94–1.06]	0.983	**1.02 [1.01–1.03]**	**0.008**	0.97 [0.92–1.02]	0.216
**CCL-3**	1.05 [0.94–1.17]	0.380	1.01 [0.98–1.03]	0.576	0.96 [0.89–1.03]	0.278
**CCL-4**	0.94 [0.88–1.00]	0.054	1.00 [0.98–1.01]	0.544	0.99 [0.96–1.01]	0.203
**TNF-α**	1.01 [0.95–1.09]	0.684	1.00 [0.99–1.02]	0.644	1.00 [1.00–1.00]	0.580
**VEGF**	0.99 [0.93–1.05]	0.707	1.00 [0.98–1.01]	0.576	**0.96 [0.93–1.00]**	**0.047**

HFNC: high flow nasal canula, NIV: non-invasive ventilation, MV: mechanical ventilation, IL-1 RA: interleukin-1 receptor antagonist, G-CSF: granulocyte colony-stimulating factor, GM-CSF: granulocyte-macrophage colony-stimulating factor, IFN-γ: interferon γ, CCL: CC-chemokine ligand, TNF-α: tumor necrosis factor α, VEGF: vascular endothelial growth factor

We found that in patients receiving NIV or MV the peak inspiratory pressure correlated with the level of IL1-RA (p = 0.018), G-CSF (p = 0.01) and IFN-γ (p = 0.042). For each unit increase in inspiratory pressure, i.e. 1 mbar, IL1-RA concentrations were estimated to increase by a factor of 1.15 [1.03–1.29] and G-CSF concentrations were estimated to increase by a factor of 1.1 [1.03–1.19]. In contrast, IFN-γ concentrations were estimated to decrease by a factor of 0.92 [0.86–0.99] for each unit increase in inspiratory pressure. In addition, fiO_2_ correlated with the serum level of CCL-7 (p = 0.008). In patients receiving high-flow nasal canula, fiO_2_ was correlated with the concentration of VEGF (p = 0.047). For each unit increase in fiO2, VEGF concentrations were estimated to decrease by a factor of 0.96.

There was no correlation of any serum peptide concentration with the ventilation modality, the occurrence of consolidations on imaging, in-hospital mortality or length of stay.

## Discussion

With over 767 million COVID-19 cases and 6.9 million deaths by the 9^th^ of July 2023 identifying risk factors for disease progression and disease severity remains essential [18].

In this prospective cohort trial, we repeatedly measured the serum concentration of peptides associated with inflammation for a period of 2 weeks in hospitalised COVID-19 patients. The serum levels were correlated with different outcomes related to disease severity, i.e. type of ventilation, level of care and radiological findings.

Here we demonstrate that increased levels of IL-1 RA were associated with increased peak pressure in patients requiring NIV or MV. The IL-1 receptor antagonist (IL-1 RA) is secreted by various immune cells and functions as an anti-inflammatory cytokine competitively inhibiting the binding of IL-1α and IL-1β to its receptor [19]. Whereas IL-1α and IL-1β serum levels did not correlate with any of the outcomes, IL-1 RA correlated with the peak pressure in patients requiring NIV or MV. In line with these results, Huang et al. demonstrated that COVID-19 patients requiring ICU treatment had higher levels of IL-1 RA compared to non-ICU patients [14]. Similarly, Yang et al. reported higher levels of IL-1 RA in severe COVID-19 patients compared to moderate cases or healthy controls [20]. IL-1 RA was suggested to be secreted as part of an endogenous anti-inflammatory response to excessive cytokine release (cytokine storm) and may therefore be elevated in patients with severe disease states. Subsequently, recombinant IL-1 RA (Anakinra) was evaluated in the treatment of COVID-19 patients. However, in two randomised controlled trials including a total of 458 COVID-19 patients, Anakinra failed to show a benefit in clinically relevant endpoints such as mortality or need for mechanical or non-invasive ventilation [21, 22].

G-CSF stimulates the proliferation of neutrophilic precursor cells and neutrophilic granulocytes. In this study, G-CSF correlated with an increased peak pressure or increased oxygen demand in COVID-19 patients requiring NIV or MV. As neutrophilia is frequently observed in blood samples and bronchoalveolar lavage samples of patients with severe COVID-19, G-CSF was suggested to promote COVID-19 pathogenesis [23, 24]. Accordingly, Kalinina et al. observed higher serum levels of G-CSF in moderate to severe COVID-19 patients compared to healthy controls [25].

CCL-7 is a chemokine that plays a crucial role in the recruitment of monocytes, neutrophils, and T-cells by facilitating migration to the site of inflammation. In this trial, CCL-7 correlated with the oxygen demand in patients requiring NIV or MV. Yang et al. demonstrated that plasma CCL-7 levels were elevated in COVID-19 patients requiring invasive ventilation or ICU treatment compared to non-ICU patients or healthy controls [26]. Additionally, elevated levels of CCL-7 were measured in bronchoalveolar lavage fluid (BALF) samples of COVID-19 patients compared to BALF samples of influenza patients [23].

IL-6 is a proinflammatory cytokine mainly produced by cells of the mononuclear phagocyte system. It mediates the synthesis of acute phase proteins and activation of lymphocytes. IL-6 was identified as a marker of disease progression and mortality in hospitalised COVID-19 patients [9, 27–30]. Here, we show that IL-6 serum levels correlated with the level of care in hospitalised COVID-19 patients. However, IL-6 was no indicator for the respective ventilation modalities in comparison to a trial published by Keddie et al. who demonstrated that IL-6 could predict the ventilatory support in COVID-19 patients requiring continuous positive airway pressure [28]. The role of IL-6 as a driver of the COVID-19 associated cytokine storm led to the use of IL-6 antagonists in COVID-19 treatment. In a meta-analysis including nine randomised controlled trials with 6428 COVID patients in total, tocilizumab reduced all-cause mortality after 28 days of treatment but did not affect clinical improvement assessed with the WHO Clinical Progression Scale [17, 31].

IFN-γ is produced by T-helper cells and NK cells and has been associated with the activation of macrophages and B lymphocytes. It plays a crucial role in the antiviral defence via initiating virus cytolysis and inhibiting viral replication [32]. In this study IFN-γ serum levels negatively correlated with the peak inspiratory pressure in patients requiring NIV or MV and were lower in ICU patients compared to general unit patients. Our results might suggest that IFN-γ levels spike at the onset of COVID and fall as the disease advances. Accordingly, IFN-γ levels were lower in severe COVID-19 patients compared to controls with moderate disease [9]. The authors suggested that SARS-CoV-2 driven depletion of T-helper cells and NK cells leads to lower IFN-γ levels in patients with severe disease. A decrease in IFN-γ levels during the course of the disease has also been observed by Gadotti et al. [32]. In contrast, Lucas et al. demonstrate that IFN-γ increased throughout the course of COVID-19 and was higher in severe cases in a longitudinal analysis of 113 patients [33].

VEGF is a growth factor produced by various cells that stimulates angiogenesis and increases endothelial permeability. In this study VEGF was negatively correlated with the fraction of inspired oxygen in patients requiring HFNC oxygen therapy. Accordingly, Gupta et al. demonstrated that VEGF declined with disease advancement in hospitalised COVID-19 patients [34]. The authors hypothesised that endothelial dysfunction leads to a decline in VEGF in severely hypoxemic patients. However, in this study the number of patients receiving HFNC was limited and results should be interpreted with caution.

The trial is subject to some limitations. This study included a limited number of patients (n = 88) and most participants received general or intermediate care, whereas only a low number of patients required ICU treatment and mechanical ventilation. In addition, there are more sensitive methods to determine cytokine concentrations in the serum, which could help to detect lower concentrations of the respective peptides in the sera. Furthermore, the serum levels of COVID-19 patients were not compared to healthy controls as the access to clinical studies was limited due to governmental restrictions. Cytokines can vary significantly among individuals as they can be affected by immunological disorders, various comorbidities and the circadian rhythm. A comparison with a healthy population would have provided more insights in this regard. In conclusion, IL1-RA, IL-6, IFN-γ, G-CSF, CCL-7 and VEGF serum levels could prove helpful as biomarkers to assess disease severity and the need for intensive care in COVID-19 patients.

## Supporting information

S1 Table Applied immunoassays and detection limits. (PDF)

S1 Fig Cytokine progression in hospitalised COVID-19 patients depending on the level of care. (PDF)
