# Supplementary material for: Ventilatory support and inflammatory peptides in hospitalised patients with COVID-19: A prospective cohort trial
Source: PLoS One. 2023 Nov 2;18(11):e0293532. doi: 10.1371/journal.pone.0293532 (PMC10621867; doi:10.1371/journal.pone.0293532)
Supplement: S1 Table — (PDF) [file pone.0293532.s001.pdf]

Epitope	Lot number	Detection limit in pg/mL
anti CCL-3	P260677	3,91
anti CCL-4	P256204	7,81
anti CCL-7	P250519	3,91
anti G-CSF	P175671	15,63
anti GM-CSF	P260758	7,81
anti IL-1 α	P254650	3,91
anti IL-1 ß	P249392	1,95
anti IL-1RA	P233771	19,53
anti IL-6	P253525	4,69
anti IL-7	P265108	3,91
anti IL-10	P248386	15,63
anti IFN-γ	P262864	4,69
anti TNF-α	P266270	7,81
anti-VEGF	P260610	15,63

IL-1 RA: interleukin-1 receptor antagonist, G-CSF: granulocyte colony-stimulating factor, GM-CSF: granulocyte-macrophage colony-stimulating factor, IFN-γ: interferon γ, CCL: CC-chemokine ligand, TNF-α: tumor necrosis factor α, VEGF: vascular endothelial growth factor
