# Supplementary material for: Ventilatory support and inflammatory peptides in hospitalised patients with COVID-19: A prospective cohort trial
Source: PLoS One. 2023 Nov 2;18(11):e0293532. doi: 10.1371/journal.pone.0293532 (PMC10621867; doi:10.1371/journal.pone.0293532)
Supplement: S1 Fig — (PDF) [file pone.0293532.s002.pdf]

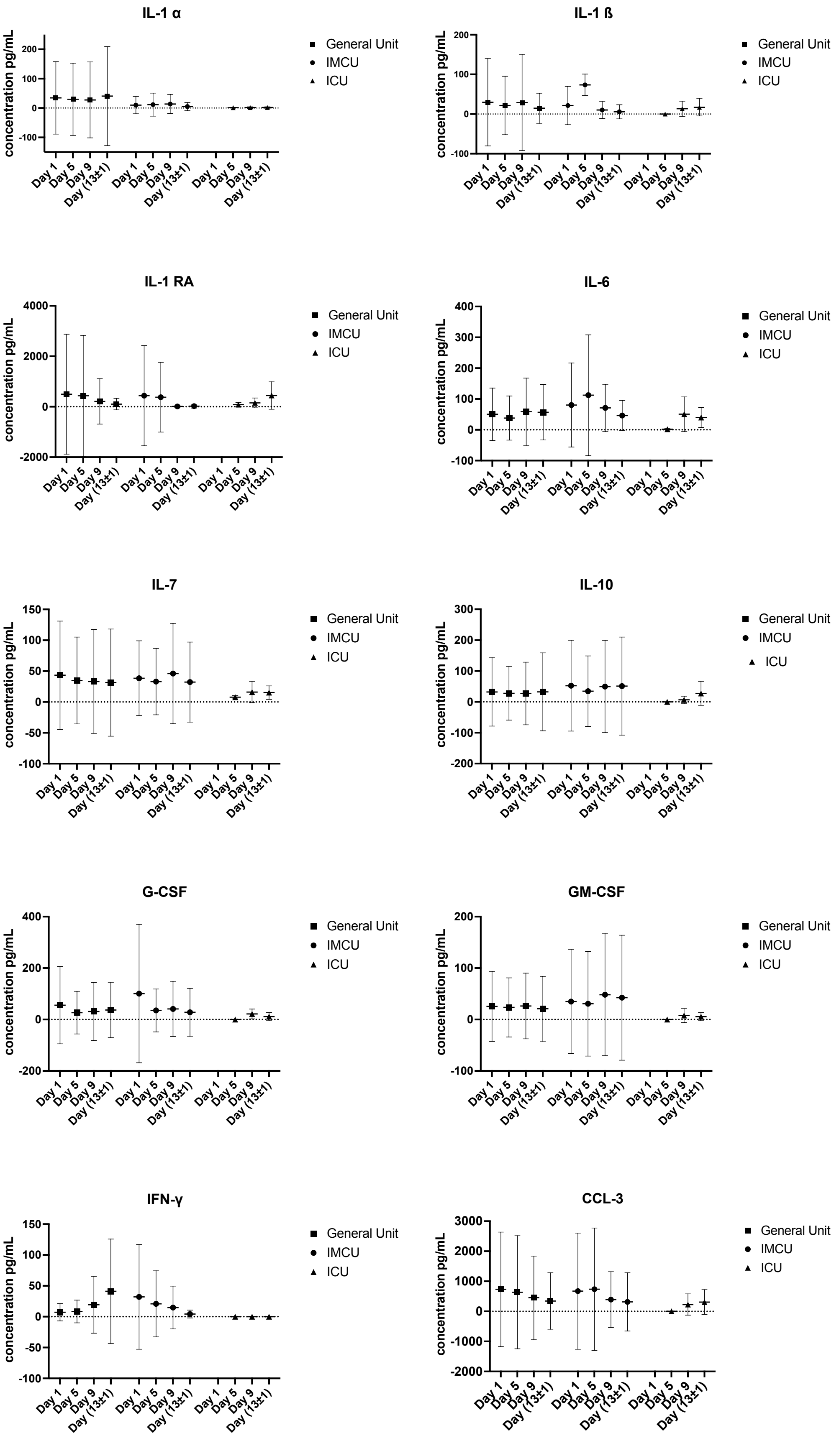
IL-1 α
IL-1 ß
IL-1 RA
IL-6
IL-7
IL-10
G-CSF
GM-CSF
IFN-γ
CCL-3
concentration pg/mL
Day 1
Day 5
Day 9
Day (13±1)
General Unit
IMCU
ICU

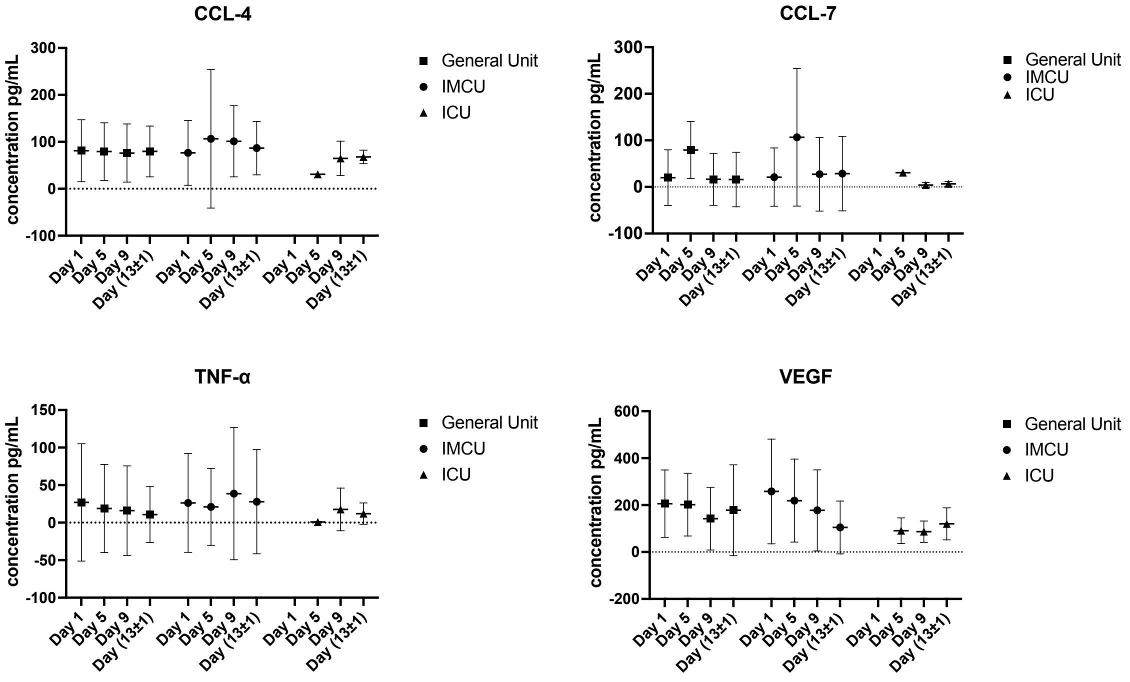

Data displayed as mean with standard deviation, IL-1 RA: interleukin-1 receptor antagonist, G-CSF: granulocyte colony-stimulating factor, GM-CSF: granulocyte-macrophage colony-stimulating factor, IFN-γ: interferon γ, CCL: CC-chemokine ligand, TNF-α: tumor necrosis factor α, VEGF: vascular endothelial growth factor, IMCU: intermediate care unit, ICU: intensive care unit
